# Sensitization profile in patients with respiratory allergic diseases: differences between conventional and molecular diagnosis (a cross-sectional study)

**DOI:** 10.1186/s12948-019-0112-4

**Published:** 2019-05-02

**Authors:** Guillermo Til-Pérez, Claudio Carnevale, Pedro Luis Sarría-Echegaray, Diego Arancibia-Tagle, Sendy Chugo-Gordillo, Manuel David Tomás-Barberán

**Affiliations:** 1 Department of Otorhinolaryngology, Hospital Universitario Son Espases, Palma de Mallorca, Spain; 2 Department of Otorhinolaryngology and Allergology, Clínica Juaneda, Palma de Mallorca, Spain

**Keywords:** Allergens, Allergic rhinitis, Asthma, Component-resolved diagnosis, Skin prick test, Polysensitization

## Abstract

**Background:**

Component-resolved diagnosis (CRD) allows to identify single molecular allergen components, and constitutes a routine practice in many allergy units. However, skin prick test (SPT) remains the technique of choice in many otorhinolaryngology departments, thus increasing the risk of using inadequate immunotherapies in patients with respiratory allergies. This study aimed to compare sensitization profiles determined by SPT and CRD in patients with respiratory allergy, and to explore the relationship between sensitization and type and severity of the respiratory disease.

**Methods:**

Cross-sectional, multicenter study of patients admitted to the Otorhinolaryngology Department due to symptoms of respiratory allergy. Extracts from various house dust mites, pollens, and molds were tested by SPT, whereas IgE against the corresponding antigens were measured by CRD.

**Results:**

The analysis included 101 patients. The sensitization profile obtained by SPT had low agreement with that of CRD, particularly to dust mite allergens (*Dermatophagoides* sp.) and pollens (*Plantago lanceolata*, *Olea europaea*, and *Cupressus sempervirens*). While SPT did not show any significant relationship between sensitization and type/severity of the respiratory disease, CRD allowed to associate Der p 1, Der f 1 and Lep d 2 sensitizations with asthma, and Der p 2, Der f 2 and Lep d 2 sensitizations with more severe symptoms of allergic rhinitis.

**Conclusions:**

Compared with SPT, CRD enables to describe a more accurate sensitization profile and to identify associations between symptoms and specific antigens. The routine use of CRD in an otorhinolaryngology setting may benefit the management of patients with respiratory allergy.

*Trial registration* IB 3108/15 (Retrospectively registered)

**Electronic supplementary material:**

The online version of this article (10.1186/s12948-019-0112-4) contains supplementary material, which is available to authorized users.

## Background

Respiratory allergies, particularly allergic rhinitis and asthma, are amongst the most common allergies in industrialized areas. They constitute a major public health and economic problem worldwide because of their prevalence, morbidity, and impact on quality of life [1–3]. Of all allergens that can elicit respiratory allergic reactions, grass pollen and house dust mites have been identified as the most prevalent [4, 5]. However, many patients are sensitized to various allergic sources, either as a result of multiple allergic responses or due to the presence of cross-reactants or panallergens [6]. This phenomenon often hinders the correct identification of the allergen source, which may ultimately lead to an inadequate diagnosis or treatment of the allergic disease [7–9].

The diagnosis of allergic diseases has been traditionally based on patient anamnesis, supported by skin prick test (SPT) and/or the identification of serum IgE in whole allergen extracts. Both methods are suitable for detecting reactivity against allergen extracts but fail to identify the specific disease-eliciting molecules. The development of the component-resolved diagnosis (CRD), also known as molecular diagnosis, has allowed to identify single molecular allergen components responsible for sensitization [10]. This feature, which allows to distinguish symptom-eliciting allergens from those attributable to cross-reactivity, has become particularly relevant since the advent of allergen-specific immunotherapy [10–12]. While CRD has been increasingly established as routine practice in many allergy units, SPT remains the technique of choice in many otorhinolaryngology departments because of its lower cost, thus increasing the risk of using inadequate immunotherapies in patients with respiratory allergies [7, 13].

Subjects living in areas with high allergy burden (e.g., Mediterranean countries) are more prone to sensitizations to minor allergens; therefore, these areas often show a higher prevalence of polysensitized patients. In this scenario, the identification of disease-eliciting molecules provided by CRD becomes even more relevant in terms of using accurate immunotherapies. In this cross-sectional study, we compared sensitization profiles determined by SPT and CRD in patients with respiratory allergy from a Mediterranean area and explored the relationship between sensitization and type and severity of the respiratory disease.

## Methods

### Study design and patients

This was a cross-sectional study of consecutive patients admitted to the otorhinolaryngology department of three hospitals in the Balearic Islands (Spain) due to symptoms of respiratory allergy between October 2012 and November 2013. The inclusion was restricted to patients of both sexes, aged between 7 and 60 years, who had been living in the same area for at least 5 years, had experienced symptoms of a respiratory allergic disease (i.e., rhinitis, asthma or both) for at least 2 years and had a positive reaction to an allergen as determined by SPT. Patients who received immunotherapy within 5 years prior to admission or who had no diagnostic tests performed were excluded from the dataset.

### Variables and endpoints

Demographic and clinical characteristics included age, sex, time from symptoms onset to diagnosis, type of respiratory allergic disease (rhinitis, asthma or rhinitis with concomitant asthma), frequency of symptoms (perennial or seasonal), and severity of the allergic disease, which was rated as mild or moderate-to-severe, based on the Spanish guidelines on the management of asthma (GEMA) [14, 15] and allergic rhinitis with impact on asthma (ARIA) [16]. Other clinical data included concomitant food allergies, and personal and/or family history of allergy. Allergen sensitization was determined using both SPT and CRD. Patients sensitized to more than one extract (SPT) or to specific IgE of more than one species (CRD) were considered polysensitized.

Skin prick test, performed according to the European Academy of Allergy and Clinical Immunology guidelines [17], was considered positive if wheals and flares were observed within 15 min, and wheal mean diameter was ≥ 3 mm. The following extracts were tested by SPT: House dust mites *Dermatophagoides pteronyssinus*, *Dermatophagoides farinae*, and *Lepidoglyphus destructor*; pollens *Olea europaea*, *Artemisia vulgaris*, *Plantago lanceolata*, *Platanus hispanica*, *Parietaria judaica*, *Salsola kali*, *Cupressus sempervirens*, and grass mixture; molds *Alternaria alternata*. Panallergen profilins, polcalcins, non-specific lipid transfer proteins (nsLTPs), and tropomyosins were also analyzed. Histamine and saline solution were used as positive and negative controls, respectively. Allergen extracts were provided by ALK-Abelló, S.A. (Madrid, Spain).

Allergen-specific IgE was quantified using chemiluminescent acridinium on the ADVIA Centaur platform (Bayer HealthCare LLC, Diagnostics Division, Tarrytown, NY, USA) as described by Petersen et al. [18]. CRD was considered positive when binding values exceeded 0.35 kU/L. The CRD analysis screened the presence of specific IgE against the following molecules: Der p 1 and 2, Der f 1 and 2, and Lep d 2; Ole e 1 and 9, Art v 1, Pla l 1, Phl p 1 and 5, Pla a 1 and 2, Par j 2, Sal k 1, Cup s 1;Alt a 1; Pho d 2, Che a 3, Pru p 3, Der p 10, and Pen a 1.

### Statistics

Quantitative variables are described as mean and standard deviation (SD) or as median and interquartile range (IQR), whereas categorical variables are presented as frequencies and percentages. Differences between groups (according to type and severity of the disease, frequency of symptoms, diagnostic method) were analyzed using the Fisher exact test or the non-parametric Wilcoxon test. Concordance between SPT and CRD methods was assessed by Cohen’s kappa coefficient (κ). Associations between demographic and clinical factors and the most prevalent sensitizations were determined by logistic regression analyses, with age, time from onset of symptoms to diagnosis, type and severity of the disease and concomitant food allergies as the independent variables. The threshold for statistical significance was established at a two-sided alpha value of 0.05. All statistical analyses were performed using the SAS 9.3 software.

## Results

### Characteristics of study patients

The analysis included 101 patients, whose demographic and clinical characteristics are summarized in Table 1. Time from onset of respiratory symptoms to diagnosis ranged from 1 to 30 years, with no differences regarding the frequency of symptoms: the mean (SD) for patients with perennial and seasonal symptoms was 7.7 (6.6) and 10.6 (8.0) years, respectively (*p *= 0.092). The most common allergic disease was rhinitis alone, followed by rhinitis with asthma. Asthma alone was present only in two patients (2%); therefore, for subsequent analyses, these patients were included in a group encompassing all patients with asthma, either alone or with concomitant rhinitis.

**Table 1:** Baseline characteristics of study patients (N = 101)

Demographic characteristics	
Age (years), *mean* (*SD*)	28.0 (14.6)
Age groups, *No.* (*%*)	
Children (≤ 14 years)	28 (27.7)
Adults (> 14 years)	73 (72.3)
Gender, *No.* (*%*)	
Female	48 (47.5)
Male	53 (52.5)
Clinical characteristics	
Time from onset of symptoms to diagnosis (years), *mean* (*SD*)	8.6 (7.1)
Frequency of symptoms, *No.* (*%*)	
Perennial	69 (68.3)
Seasonal	32 (31.7)
Respiratory allergic disease^a^, *No.* (*%*)	
Rhinitis	74 (74.7)
Asthma	2 (2.0)
Rhinitis and asthma	23 (23.2)
Severity of rhinitis, *No.* (*%*)	
Mild intermittent	28 (29.5)
Mild persistent	31 (32.6)
Moderate-severe intermittent	7 (7.4)
Moderate-severe persistent	29 (30.5)
Severity of asthma, *No.* (*%*)	
Mild intermittent	14 (56.0)
Mild persistent	4 (16.0)
Moderate intermittent	0 (0.0)
Moderate persistent	7 (28.0)
Food allergy, *No.* (*%*)	7 (6.9)
History of previous allergy, *No.* (*%*)	9 (9.0)
Family history of allergy, *No.* (*%*)	41 (41.0)

^a^Calculated over 99 patients

### Sensitization profile

Table 2 shows the sensitization profiles obtained with each diagnostic method. Most prevalent sensitizations, present in more than 20% of patients, were to *D. pteronyssinus* (Der p 1 and 2), *D. farinae* (Der f 1 and 2), *L. destructor* (Lep d 2), *Olea europaea* (Ole e 1), and *Phleum pratense* (Phl p 1) allergens. The percentage of sensitized patients was significantly higher with SPT than CRD for Der p 1 and 2, Der f 1 and 2, Lep d 2, Ole e 1, Pla l 1, and Sal k 1. However, Alt a 1 and Pru p 3 showed a lower percentage of sensitized subjects when SPT was used compared with CRD. Overall, 91 (93.8%) and 72 (79.1%) patients were polysensitized according to SPT and CRD tests, respectively. The results regarding the quantitative determination of allergen-specific IgE are summarized in Additional file 1: Table S1.

**Table 2:** Profile of sensitization regarding the diagnostic method, *No* (*%*)

	SPT	CRD	*p*
House dust mites			
*Dermatophagoides pteronyssinus*	69 (68.3)		*0.031*
Der p 1		44 (44.4)	
Der p 2		53 (53.5)	
Der p 1 or 2		58 (58.6)	
*Dermatophagoides farinae*	70 (69.3)		*0.021*
Der f 1		41 (41.4)	
Der f 2		52 (52.5)	
Der f 1 or 2		59 (59.6)	
*Lepidoglyphus destructor*	42 (41.6)		*< 0.001*
Lep d 2		21 (21.2)	
Pollens			
*Olea europaea*	57 (56.4)		*0.019*
Ole e 1		43 (44.3)	
Ole e 9		2 (2.0)	
*Artemisia vulgaris*	8 (7.9)		1.000
Art v 1		9 (9.2)	
*Plantago lanceolata*	25 (24.8)		*< 0.001*
Pla l 1		5 (5.1)	
Mixture of grasses	45 (44.6)		0.302
Phl p 1		34 (34.3)	
Phl p 5		12 (12.1)	
Phl p 1 or 5		38 (38.4)	
*Platanus hispanica*	9 (8.9)		0.754
Pla a 1 + 2		6 (6.1)	
*Parietaria judaica*	18 (17.8)		0.754
Par j 2		16 (16.3)	
*Salsola kali*	10 (9.9)		*0.039*
Sal k 1		3 (3.0)	
*Cupressus sempervirens*	15 (14.9)		0.125
Cup s 1		9 (9.2)	
Molds			
*Alternaria alternata*	12 (11.9)		*0.039*
Alt a 1		18 (18.2)	
Panallergens			
Profilins	3 (3.0)		0.500
Pho d 2		1 (1.0)	
Polcalcins	5 (5.0)		0.125
Che a 3		1 (1.0)	
LTPs	4 (4.0)		*0.031*
Pru p 3		10 (10.1)	
Tropomyosins	NA		NA
Der p 10		3 (3.0)	
Pen a 1		4 (4.0)	

*p* values in italics correspond to significant differences at a bilateral alpha value of 0.05

*CRD* component-resolved diagnostics, *LTPs* lipid transfer proteins, *NA* not available, *SPT* skin prick test

Cohen’s kappa of SPT vs. CRD for allergens with significant differences in the sensitization profile revealed low agreement between both diagnosis methods: κ = 0.12 (95% CI − 0.10, 0.35) for *D. pteronyssinus* vs. Der p 1 and 2; κ = − 0.05 (95% CI − 0.12, 0.01) for *D. farinae* vs. Der f 1 and 2;κ = 0.05 (95% CI − 0.08, 0.19) for *L. destructor* vs. Lep d 2; κ = − 0.01 (95% CI − 0.20, 0.17) for *O. europaea* vs. Ole e 1 and 9; and κ = 0.07 (95% CI − 0.09, 0.23) for *P. lanceolata* vs. Pla l 1 and 2. Cohen’s kappa could not be determined for Sal k 1 and Alt a 1.

### Association between allergens and diseases

Table 3 summarizes the association between allergen sensitizations and type of respiratory allergic disease. SPT did not reveal significant associations, except for the extract of *P. lanceolata*, the sensitization to which was more frequent in patients with rhinitis than those with asthma (with or without concomitant rhinitis). On the other hand, sensitization to house dust mite allergens Der p 1, Der f 1 and Lep d 2 were significantly more frequent in patients with asthma (with or without concomitant rhinitis) compared to patients with rhinitis alone (Table 3).

**Table 3:** Associations between allergens and disease diagnosed by SPT and CRD

	SPT			CRD		
	Asthma^a^ (N = 25)	Rhinitis (N = 74)	*p*	Asthma^a^ (N = 25)	Rhinitis (N = 74)	*p*
House dust mites						
*Dermatophagoides pteronyssinus*	19 (95.0)	49 (94.2)	1.000			
Der p 1				15 (65.2)	28 (37.8)	*0.037*
Der p 2				16 (69.6)	36 (48.6)	0.118
*Dermatophagoides farinae*	19 (100.0)	50 (96.2)	1.000			
Der f 1				15 (65.2)	25 (33.8)	*0.010*
Der f 2				15 (65.2)	36 (48.6)	0.232
*Lepidoglyphus destructor*	14 (100)	27 (87.1)	0.294			
Lep d 2				10 (43.5)	10 (13.5)	*0.004*
Pollens						
*Olea europaea*	17 (94.4)	38 (92.7)	1.000			
Ole e 1				13 (59.1)	28 (38.4)	0.106
Ole e 9				1 (4.4)	1 (1.4)	0.420
*Artemisia vulgaris*	1 (50.0)	7 (77.8)	0.491			
Art v 1				1 (4.4)	8 (11.0)	0.710
*Plantago lanceolata*	4 (57.1)	21 (95.5)	*0.034*			
Pla l 1				0 (0.0)	5 (6.8)	0.607
*Phleum pratense*	12 (92.3)	32 (100)	0.289			
Phl p 1				8 (34.8)	25 (33.8)	0.871
Phl p 5				1 (4.4)	11 (14.9)	0.374
*Platanus hispanica*	4 (100.0)	5 (83.3)	1.000			
Pla a 1 + 2				1 (4.4)	4 (5.4)	1.000
*Parietaria judaica*	3 (100.0)	15 (93.8)	1.000			
Par j 2				4 (17.4)	12 (16.4)	1.000
*Salsola kali*	1 (100.0)	9 (100.0)	–			
Sal k 1				0 (0.0)	3 (4.1)	1.000
*Cupressus sempervirens*	4 (100.0)	11 (91.7)	1.000			
Cup s 1				2 (9.1)	7 (9.5)	1.000
Molds						
*Alternaria alternata*	3 (100.0)	9 (100.0)	–			
Alt a 1				3 (13.0)	15 (20.3)	0.801
Panallergens						
Profilins						
Pho d 2	2 (66.7)	1 (33.3)	1.000			
Polcalcins						
Che a 3	0 (0.0)	5 (83.3)	0.286			
LTPs						
Pru p 3	1 (50.0)	3 (100.0)	0.400			
Tropomyosins						
Der p 10				1 (4.5)	2 (2.7)	*0.041*
Pen a 1				1 (4.4)	3 (4.1)	1.000

*p* values in italics correspond to significant differences at a bilateral alpha value of 0.05

Positive sensitizations. *No.* (*%*)

*CRD* component-resolved diagnostics, *LTPs* lipid transfer proteins, *NA* not available, *SPT* skin prick test

^a^With or without concomitant rhinitis

When analyzing disease severity regarding sensitization to specific molecules, sensitization to Der p 2, Der f 2, and Lep d 2 appeared to be associated with moderate-to-severe rhinitis (Table 4). This trend was not observed in the corresponding primary antigens of *Dermatophagoides* sp. Der p 1 and Der f 1.

**Table 4:** Associations between allergens and severity disease diagnosed by component-resolved diagnostics

	Asthma^a^			Rhinitis		
	Mild (N = 18)	Moderate-severe (N = 7)	*p*	Mild (N = 59)	Moderate-severe (N = 36)	*p*
House dust mites						
Der p 1	11 (68.8)	4 (57.1)	0.657	25 (43.1)	17 (47.2)	0.831
Der p 2	12 (75.0)	4 (57.1)	0.626	25 (43.1)	26 (72.2)	*0.010*
Der f 1	11 (68.8)	4 (57.1)	0.657	23 (39.7)	16 (44.4)	0.672
Der f 2	12 (75.0)	3 (42.9)	0.182	25 (43.1)	25 (69.4)	*0.019*
Lep d 2	7 (43.8)	3 (42.9)	1.000	7 (12.1)	12 (33.3)	*0.018*
Pollens						
Ole e 1	9 (60.0)	4 (57.1)	1.000	25 (43.1)	14 (41.2)	1.000
Ole e 9	1 (6.3)	0 (0.0)	1.000	1 (1.7)	1 (2.8)	1.000
Art v 1	1 (6.3)	0 (0.0)	1.000	5 (8.8)	4 (11.1)	0.731
Pla l 1	0 (0.0)	0 (0.0)	–	2 (3.4)	3 (8.3)	0.368
Phl p 1	7 (43.8)	1 (14.3)	0.345	17 (29.3)	16 (44.4)	0.183
Phl p 5	1 (6.3)	0 (0.0)	1.000	6 (10.3)	5 (13.9)	0.744
Pla a 1 + 2	0 (0.0)	1 (14.3)	0.304	4 (6.9)	1 (2.8)	0.646
Par j 2	2 (12.5)	2 (28.6)	0.557	8 (14)	7 (19.4)	0.567
Sal k 1	0 (0.0)	0 (0.0)	–	1 (1.7)	2 (5.6)	0.556
Cup s 1	1 (6.3)	1 (16.7)	0.480	6 (10.5)	3 (8.3)	1.000
Molds						
Alt a 1	2 (12.5)	1 (14.3)	1.000	9 (15.5)	9 (25.0)	0.289
Panallergens						
Pho d 2						
Che a 3						
Pru p 3						
Der p 10	0 (0.0)	1 (16.7)	0.273	0 (0.0)	2 (5.6)	0.147
Pen a 1	1 (6.3)	0 (0.0)	1.000	2 (3.4)	2 (5.7)	0.630

*p* values in italics correspond to significant differences at a bilateral alpha value of 0.05

Positive sensitizations, *No.* (*%*)

^a^With or without concomitant rhinitis

### Demographic and clinical factors influencing sensitization

The results of the multivariate analysis to predict sensitization to each of the most prevalent antigens based on clinical and demographic characteristics are summarized in Table 5. The analysis failed to yield a multivariate model to predict sensitization to plant antigens Ole e 1 and Phl p 1. On the other hand, either the type or severity of the disease significantly contributed to sensitization toall house dust mite allergens included in the analysis (Table 5).

**Table 5:** Factors associated with the sensitization to allergens

	Der p 1		Der p 2		Der f 1		Der f 2		Lep d 2		Ole e 1		Phl p 1	
	OR (95% CI)	*p*	OR (95% CI)	*p*	OR (95% CI)	*p*	OR (95% CI)	*p*	OR (95% CI)	*p*	OR (95% CI)	*p*	OR (95% CI)	*p*
Age	0.47 (0.19–1.15)	0.097	0.65 (0.27–1.58)	0.339	0.48 (0.20–1.17)	0.107	0.75 (0.31–1.81)	0.523	0.44 (0.16–1.20)	0.107	0.46 (0.19–1.14)	0.093	2.44 (0.88–6.79)	0.086
Time from onset of symptoms to diagnosis	1.04 (0.98–1.0)	0.184	1.03 (0.97–1.09)	0.302	1.02 (0.97–1.08)	0.455	1.04 (0.98–1.10)	0.227	0.98 (0.91–1.05)	0.510	1.02 (0.96–1.07)	0.590	1.04 (0.98–1.10)	0.194
Disease	2.87 (1.07–7.20)	*0.036*	2.26 (0.83–6.19)	0.112	3.43 (1.27–9.26)	*0.015*	1.85 (0.69–4.93)	0.220	5.33 (1.83–15.6)	*0.002*	2.14 (0.80–5.74)	0.129	1.12 (0.41–3.02)	0.823
Severity	1.47 (0.62–3.50)	0.379	4.00 (1.62–9.89)	*0.003*	1.57 (0.66–3.74)	0.307	4.00 (1.62–9.89)	*0.003*	5.98 (1.76–20.3)	*0.004*	1.07 (0.45–2.56)	0.877	2.08 (0.86–5.06)	0.106
Concomitant food allergy	2.72 (0.47–15.6)	0.262	0.88 (0.17–4.58)	0.877	3.11 (0.54–7.9)	0.203	0.44 (0.08–2.52)	0.355	1.92 (0.33–11.3)	0.470	7.16 (0.80–63.8)	0.078	0.36 (0.04–3.19)	0.357

*p* values in italics correspond to significant differences at a bilateral alpha value of 0.05

*CI* confidence interval, *OR* odds ratio

## Discussion

In this cross-sectional study, we found that the sensitization profile of patients with respiratory allergy obtained by the traditional SPT has low agreement with that of CRD, particularly to dust mite allergens and pollens, such as *P. lanceolata*, *O. europaea*, and *C. sempervirens*. Furthermore, whereas SPT failed to reflect a significant relationship between sensitization and type/severity of the respiratory disease, CRD allowed to identify Der p 1, Der f 1 and Lep d 2 as antigens more frequently associated with asthma, and Der p 2, Der f 2 and Lep d 2 as antigens associated with more severe symptoms in patients with allergic rhinitis.

The low agreement between SPT and CRD observed in our analysis is consistent with that reported by previous authors [19, 20]. One of the major consequences of inconsistency in sensitization profiles is the inaccurate prescription of immunotherapy for patients with respiratory symptoms. In this regard, some authors have observed that the choice of immunotherapy may vary in about 55% of cases after comparing SPT and CRD sensitization profiles [7, 13] and that respondent rates increase when immunotherapy is used based on CRD results [9]. In this study, the number of sensitizations diagnosed by CRD was generally lower than those identified by SPT, being *P. lanceolata* the allergen that showed the greater difference (prevalence was 80% lower with CRD than with SPT). This finding was in line with that reported by Orovitg et al. [20]. and confirms Pla l 1 as a minor allergen eliciting respiratory allergic diseases in Mediterranean areas. Although this significant difference in sensitization to *P. lanceolata* could be attributed to the presence of panallergens (e.g., profilin), the small number of patients sensitized to panallergens in our study prevented further analyses in this regard. Other pollens with a strong presence in Mediterranean areas, such as *Olea* sp. [21], followed the same trend, albeit in a lesser extent. Of note, the differences between SPT and CRD for *Olea* sp. pollens found in our cohort were similar than those reported by previous authors in areas with higher prevalence of these pollens [20, 22]. Unlike plant antigens, the proportion of patients sensitized to LTPs (Pru p 3) and *A. alternata* were higher with CRD than SPT. Although our analysis does not allow us to draw strong conclusions in this regard, the cross-reactivity described for Art v 3 and Pru p 3 may have contributed to this observation [20].

The association between sensitization to specific allergens and the occurrence of respiratory diseases such as asthma and rhinitis has motivated the use of immunotherapy in those patients [23, 24]. In fact, allergen-specific immunotherapy is currently the only causal treatment for allergic rhinitis that can reduce its symptoms and the need for medical therapies [25]. However, in routine clinical practice, these therapies are often based on SPT diagnoses, assuming sensitization to main allergens. In our cohort, sensitization profiles determined by SPT did not reveal significant a relationship between sensitization and either the type of respiratory allergy or symptom severity. Conversely, CRD allowed to associate sensitizations to Der p 1, Der f 1 and Lep d 2 with asthma (with or without concomitant rhinitis), and sensitizations to Der p 2, Der f 2 and Lep d 2 with moderate-to-severe diseases in patients with rhinitis. Consistently with this finding, the multivariate analysis showed that the type of disease significantly contributed to sensitization to group 1 (Der p 1 and Der f 1) house dust mite allergens, whereas the severity of the disease significantly influenced sensitization to group 2 (Der p 2 and Der f 2) house dust mite allergens; sensitization to Lep d 2 was associated with both the type and severity of the disease. Similar relationships have been reported between various pollens and the type of respiratory disease [22, 26, 27]; however, to our knowledge, this is the first study showing a relationship between disease type/severity and house dust mite allergens. Although evidence regarding these specific relationships is still weak, consistent findings that specific antigens are associated with clinical characteristics of the respiratory disease strengthen the importance of using CRD to assess the sensitization profile of patients with respiratory allergy.

The main limitation of our study was its cross-sectional design, which restricted data collection to a particular period of the year for each patient, thus losing sight of changes in sensitization profiles caused by variations in antigen prevalence throughout the seasons. Although most patients in our cohort had perennial symptoms (and, therefore, were expected to show consistent sensitization profiles throughout the year), sensitization to pollens has a high seasonal variability in our area [21]. Another limitation of our results was the low prevalence of panallergens found in our study cohort. The presence of these allergens, which is commonly higher in other studies [20, 22, 27], has been considered a key benefit of CRD and may increase the differences between the two types of diagnosis.

## Conclusions

Our results emphasize the limitations of SPT to determine sensitization profiles of patients with respiratory allergy in an otorhinolaryngology setting. Although the relationship between symptoms and specific antigens is still poorly understood, the ability of CRD to depict more accurate sensitization profiles and identify candidates to symptom-triggering antigens indicates that the routine use of CRD in this setting may provide remarkable benefits for the management of patients with respiratory allergy. The assessment of the cost-effectiveness of this approach in future studies is warranted.

## Additional file

**Additional file 1: Table S1.** Quantitative determination of allergen-specific IgE.

## Abbreviations

CRD: component-resolved diagnosis
nsLTPs: non-specific lipid transfer proteins
SPT: skin prick test
