# Supplementary material for: Sensitization profile in patients with respiratory allergic diseases: differences between conventional and molecular diagnosis (a cross-sectional study)
Source: Clin Mol Allergy. 2019 May 2;17:8. doi: 10.1186/s12948-019-0112-4 (PMC6495638; doi:10.1186/s12948-019-0112-4)
Supplement: Supplementary file 1 — Additional file 1: Table S1. Quantitative determination of allergen-specific IgE. [file 12948_2019_112_MOESM1_ESM.docx]

# Sensitization Profile in Patients with Respiratory Allergic Diseases: Differences Between Conventional and Molecular Diagnosis (a cross-sectional study)

Additional file 1

| **Table S1.** Quantitative determination of allergen-specific IgE | | |
| --- | --- | --- |
|  | **n** | **Median (Q1-Q3)** |
| **House dust mites** |  |  |
| Der f 1 | 41 | 4.27 (0.95-7.08) |
| Der f 2 | 52 | 57.95 (12.99-159.41) |
| Der p 1 | 44 | 7.59 (3.09-16.64) |
| Der p 2 | 53 | 66.63 (14.12-15138) |
| Blo t 5 | 4 | 9.16 (3.44-12.62) |
| Lep d 2 | 21 | 5.64 (2.02-10.31) |
| Der p 10 | 3 | 15.10 (0.50-25.17) |
| Pen a 1 | 4 | 7.00 (0.67-23.85) |
| **Pollen** |  |  |
| Art v 1 | 9 | 2.27 (1.04-4.09) |
| Bet v 1 | 2 | 4.23 (1.04-7.42) |
| Cup s 1 | 9 | 2.58 (1.53-4.01) |
| Ole e 1 | 43 | 3.83 (0.93-9.60) |
| Ole e 9 | 2 | 19.45 (5.37-33.53) |
| Phl p 1 | 34 | 2.26 (0.80-5.19) |
| Phl p 5 | 12 | 3.89 (1.74-14.65) |
| Pla l 5 | 5 | 1.00 (0.59-1.19) |
| Sal k 5 | 3 | 1.28 (0.74-5.67) |
| Pla a 1+2 | 6 | 0.68 (0.62-0.71) |
| Par j 2 | 16 | 7.73 (1.05-52.08) |
| **Molds** |  |  |
| Alt a 1 | 18 | 2.52 (1.10-15.48) |
| **Panallergens** |  |  |
| Pho d 2 | 1 | 5.11 |
| Che a 3 | 1 | 1.63 |
| Pru p 3 | 10 | 1.80 (1.19-5.39) |
